# Twist3 is required for dedifferentiation during extraocular muscle regeneration in adult zebrafish

**DOI:** 10.1371/journal.pone.0231963

**Published:** 2020-04-22

**Authors:** Yi Zhao, Ke’ale W. Louie, Christina F. Tingle, Cuilee Sha, Curtis J. Heisel, Shelby P. Unsworth, Phillip E. Kish, Alon Kahana

**Affiliations:** 1 Department of Ophthalmology and Visual Sciences, Kellogg Eye Center, University of Michigan, Ann Arbor, Michigan, United States of America; 2 Department of Biologic and Materials Sciences, School of Dentistry, University of Michigan, Ann Arbor, Michigan, United States of America; East Carolina University, UNITED STATES

## Abstract

Severely damaged adult zebrafish extraocular muscles (EOMs) regenerate through dedifferentiation of residual myocytes involving a muscle-to-mesenchyme transition. Members of the Twist family of basic helix-loop-helix transcription factors (TFs) are key regulators of the epithelial-mesenchymal transition (EMT) and are also involved in craniofacial development in humans and animal models. During zebrafish embryogenesis, twist family members (twist1a, twist1b, twist2, and twist3) function to regulate craniofacial skeletal development. Because of their roles as master regulators of stem cell biology, we hypothesized that twist TFs regulate adult EOM repair and regeneration. In this study, utilizing an adult zebrafish EOM regeneration model, we demonstrate that inhibiting twist3 function using translation-blocking morpholino oligonucleotides (MOs) impairs muscle regeneration by reducing myocyte dedifferentiation and proliferation in the regenerating muscle. This supports our hypothesis that twist TFs are involved in the early steps of dedifferentiation and highlights the importance of twist3 during EOM regeneration.

## Introduction

Skeletal muscle injuries and degenerative conditions are common, debilitating, and significant causes of morbidity and mortality worldwide [1, 2]. Despite the pervasiveness of injury, mammalian muscle repair is limited by the extent of tissue damage and restricted by the amount of resident stem cells (*i*.*e*. satellite cells) available for tissue replacement [3]. This differs from non-mammalian vertebrates such as zebrafish which robustly regenerate both skeletal and cardiac muscle as well as other tissues including retina, spinal cord, liver, and fin [4–8]. Such extensive repair, or rather whole tissue regeneration, relies less on the activation of resident stem cells and more on cell reprogramming and dedifferentiation [9, 10]. Understanding the mechanisms underlying adult *de novo* muscle regeneration in model vertebrates thus represents a topic with widespread clinical therapeutic implications [2, 11]. Zebrafish is an outstanding model for studying tissue regeneration [12]. Our lab has developed a novel zebrafish-based system to study regeneration of extraocular muscles (EOMs)—a form of skeletal muscle whose regeneration is driven by myocyte dedifferentiation with no significant contribution from satellite cells [10].

The twist family of basic helix-loop-helix (bHLH) transcription factors (TFs) represent an evolutionarily conserved family of proteins that regulate stem cells during both embryonic development [13–15] and adult progenitor cell maintenance [16]. Twist orthologs are also known regulators of muscle formation and regeneration in *Drosophila* [17, 18], muscle stem cells during mouse development [19], and skeletal muscle repair in adult mice [20, 21]. Zebrafish have 4 twist homologs—twist1a, twist1b, twist2, and twist3—which are orthologs of mammalian twist1 and twist2 [22] and are necessary for proper craniofacial development [23]. The prevailing belief that regeneration is a recapitulation of embryonic development led us to hypothesize that twist TFs are involved in adult extraocular muscle (EOM) regeneration. We therefore sought to identify which zebrafish twist homologs participate in the regeneration process and at what timepoint.

Utilizing our established regeneration model, we report that twist3 is the sole twist TF required for EOM regeneration in adult zebrafish. Knockdown of twist3 significantly impairs muscle regeneration by decreasing myofiber dedifferentiation and cell proliferation post-injury. These findings suggest that twist3 plays an early role during the myocyte dedifferentiation process that precedes cell cycle re-entry. Additionally, knockdown of other zebrafish twist homologs (*i*.*e*. twist1a, twist1b, and twist2) did not affect regeneration parameters, thereby suggesting fundamental differences between embryonic development and adult muscle regeneration in zebrafish.

## Methods

### Zebrafish (*danio rerio*) rearing and surgeries

All animal work was performed in compliance with the ARVO Statement for the Use of Animals in Ophthalmic and Vision Research and approved by the University of Michigan Committee on the Use and Care of Animals, protocol 06034. Sexually mature adult (4–18 month-old) zebrafish were spawned in our fish facility and raised per standard protocol [24] at 28 °C with a 14-h light/10-h dark alternating cycle.

Adult zebrafish were anesthetized using 0.05% tricaine methanosulfate (Tricaine-S; Western Chemical, Ferndale, WA) with 0.05% sodium bicarbonate buffer and about 50% of the lateral rectus (LR) muscle was surgically excised, i.e. myectomy. The length of the regenerating muscle was quantified by craniectomy as described previously [25]. Regeneration is represented as the relative size of the injured LR muscle normalized to the length of the uninjured LR muscle (representing 100%). All experiments were performed using 5 fish per experimental group and/or time point, unless stated otherwise in the text and/or figure legend.

### Twist TFs customized antibody

Polyclonal rabbit antibodies to twist proteins (twist1a, twist1b, twist2, and twist3) were custom produced by Hitag Biotechnology, Lda, Cantanhede Portugal. Briefly, codon optimized 6x-His tagged proteins were expressed in bacteria, the protein was purified using multiple steps and the His-tag removed with tobacco etch virus (TEV) protease. Rabbits were immunized with the purified proteins and the resulting sera purified by Protein A affinity chromatography. Twist 3 antibodies were further affinity purified using His-tagged zebrafish twist3 over-expressed protein (expressed in HEK293 cells) and affinity purified using cobalt-IMAC chromatography. The purified tagged twist 3 protein was coupled to a MicroLink™ Protein Coupling Kit column (Pierce, Rockford, IL) and the antibodies purified according to the manufacturer’s instructions. Antibodies were validated for Western blot specificity utilizing mammalian over-expression plasmids transiently transfected into HEK293 cells with Lipofectamine 2000 (Invitrogen, Carlsbad CA).

### Protein extraction, immunoprecipitation, and Western blots

For embryo protein extraction, dechorionated and deyolked zebrafish embryos were pooled and homogenized in RIPA lysis buffer (Cell Signaling Technology, Danvers, MA) in a ratio of 100 μL RIPA/30 embryos. For adult LR muscle protein extraction, transgenic Tg(α-actin::EGFP) fish were used to visualize the muscles. Muscle tissue were collected as previously described [26]. Injured or uninjured LR were pooled in denaturing buffer (1% SDA, 5mM EDTA, 10mM beta-mercaptoethanol, Protease inhibitors, 15 U/ml DNase1) in a ratio of 30 muscle/100 μL buffer and homogenized by passing lysis through a 27-gauge needle attached to a 1 mL syringe. Heat samples to 95 °C for 5 min to denature and centrifuge to collect supernatant. Protein concentrations were determined by BCA assay (Thermo Scientific, 23227).

Same amount of protein (~100 uL) were diluted by non-denaturing buffer (20 mM Tris HCl, 137 mM NaCl, 10% glyceral, 1% NP-40, 2mM EDTA) to make a total volume of 1mL and proceed with the immunoprecipitation.

Protein samples were incubated with customized twist antibodies 1:100 at 4°C with continuous mixing overnight. The following day, 200 μL washed PureProteome™ Protein A Magnetic Beads (Cat. No. LSKMAGA02, Lot 2674904A, Germany) were added per 1 mL of sample, and this was allowed to incubate for 45–60 minutes at 4°C with continuous mixing. The supernatant was removed, and the beads were washed 3x5min with 0.1% PBS-Tween20. After the last wash, the buffer was removed, and Laemmli Sample buffer was added proportional to number of muscles collected. This was boiled at 90°C for 10 minutes before removing the solution for use in Western blots.

Anti-Tubg1 (1:1000, Sigma, T5326), anti-beta actin (1:30,000, Santa Cruz, sc-47778 HRP), anti-p-histone H3 (1:1000, Cell Signaling Technology, 9701), and customized twist TFs antibodies were used to detect protein.

### Drug treatments

SU5402 (Selleckchem, Houston, TX) was dissolved in DMSO as a 17 mM stock and added to fish water at a final concentration of 17 μM as described [27], tanks were kept in the dark. Up to 5 fish were treated in 250 mL of water, tanks were maintained at 28.5°C, and drug solutions were replaced every 24 h. Drug treatments were performed 24 h before surgery and no significant mortality was noted.

### Morpholino oligonucleotide injection and electroporation

Microinjection of morpholino oligonucleotides (MOs; Gene-Tools, LLC, Philomath, OR)—a widely used technique to perform knockdown experiments in zebrafish [29–31]—was used. To knockdown genes in adult EOMs, lissamine-tagged MOs were directly microinjected into the right LR muscle of Tg(α-actin::EGFP) adult fish, followed by square-wave electroporation (6 to 10 pulses at 48 V/cm, BTX ECM830 electroporator; Harvard Apparatus, Holliston, MA). Microinjections were performed 4 h prior to LR injury, and MO uptake was confirmed via lissamine fluorescence prior to myectomy. No mortality was detected during the experimental process. MO sequences are listed in Table 1; a standard control MO targeting a mutated splice site of human β-globin mRNA was injected for each experiment as negative control.

**Table 1:** Sequences for morpholino oligonucleotides.

Name	Sequence
**Standard control**	5’-CCTCTTACCTCAGTTACAATTTATA-3’
***Twist1a*-MO**	5’-GTGCATCGCCTCTTCCTCAAACATC-3’
***Twist1b*-MO**	5’-CGGGCTCTTCGGGCATCTCGCTTAA-3’
***Twist2*-MO**	5’-AATACGATCTCCACTTTTGGTTCCG-3’
***Twist3*-MO**	5’-TCCACAAGTCTGTTCCTCTCGCATG-3’

### EdU incorporation assays

Cellular proliferation was assessed by intra-peritoneal (IP) injections of 5-ethynyl-2’-deoxyuridine (EdU) and standard detection methods [10]. Fish were anesthetized and injected with EdU (20 μL, 10 mM EdU in PBS) at 20 hpi or 44 hpi and sacrificed 4 h later (24 hpi or 48 hpi). For each experiment, 3 fish per group were analyzed. The injured muscle of each fish was analyzed with both EdU-positive and total (DAPI-positive) nuclei counted from 3 nonconsecutive sections per muscle. Representative sections had approximately 1800 total nuclei (range 812–3016) per muscle. Cell proliferation is represented as the percentage of EdU-positive nuclei in the injured muscle.

### Specimen processing

Zebrafish heads were excised and decalcified using Magic-EDTA (10% EDTA, saturated ammonium sulfate in PBS, Ph7.4) for 3 days. Decalcified tissues were fixed in 4% paraformaldehyde (PFA) overnight at 4°C. Decalcified and fixed tissues were cryoprotecteded with 20% sucrose in PBS, embedded in OCT (Fisher Scientific), frozen, and evaluated microscopically using coronal frozen sections (12 μm) as described previously [10].

### Statistics

Comparisons between 2 groups were analyzed by Student t-test (*p < 0.05; **p < 0.01; ***p < 0.001). When more than 2 groups were compared, one-way analysis of variance (ANOVA, P < 0.05) followed by Newman-Keuls multiple comparisons test (p < 0.05) was performed. Thus, in the time course experiments, differences between fish groups for each time point were analyzed by Student t-test and differences among time points for each fish group were analyzed by ANOVA. All tests were performed using the statistical software Prism 6.03 (GraphPad, LaJolla, CA, USA).

## Results

### Inhibition of twist3 impairs adult zebrafish EOM regeneration

**Fig 1:**
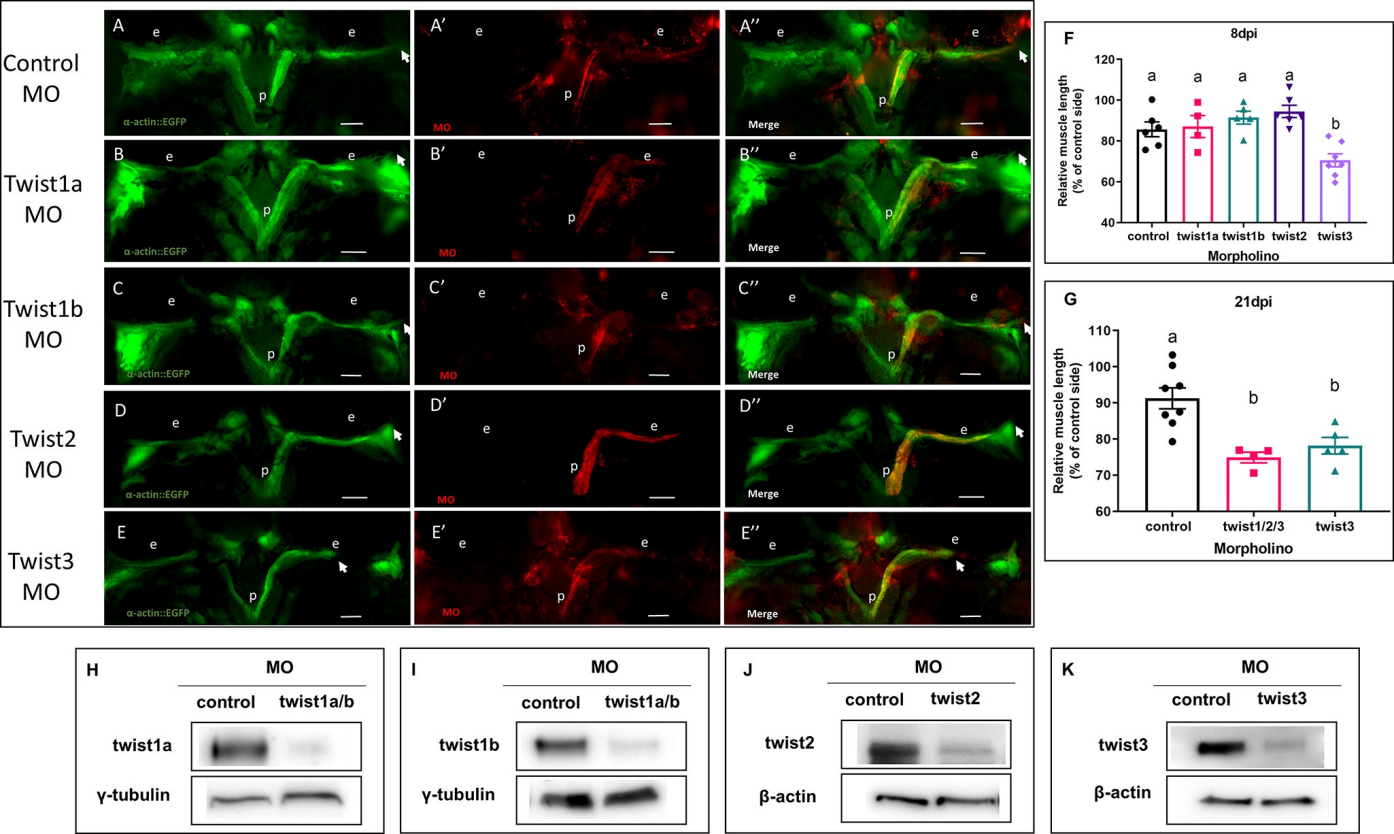
Inhibition of Twist3, but not other Twist TFs, impairs muscle regeneration. To knock down Twist TFs, lissamine-tagged MOs (red) against twist 1a, 1b, 2, and 3 were microinjected into Tg(*α-actin*::*EGFP*) (green) fish muscles 4 h prior to myectomy. (A-E'') MO injected fish were shown. (H-J) Knockdown effect of twist 1a/b, and twist2 MO was validated in embryos by Western blot. (K) Knockdown effect of twist3 MO was validated in EOM by Western blot. (F) The length of regenerating muscle was measured as described; values are averages ±SEM (one-way ANOVA, p<0.05 between group a and b, control:n = 6, twist1a: n = 4, twist1b: n = 5, twist2: n = 6, twist3: n = 7). (G) Twist3 MO inhibits but does not delay EOM regeneration; mixture of all 4 Twist TFs MOs or single control and twist3 MO were microinjected into α-actin-EGFP fish muscles 4 h prior to myectomy. The length of regenerating muscle was measured as described; values are averages ±SEM (one-way ANOVA, p<0.05 between group a and b, control: n = 8, twist1/2/3: n = 4, twist3: n = 5). White arrows marked the growing end of the regenerating muscle. p, pituitary; e, eye; scale bar: 250μm.

Twist TFs are expressed during zebrafish embryogenesis and regulate craniofacial skeletal development [23, 28]. They are also known to be master regulators of stem cells [29, 30]. Given the need to generate dedifferentiated myoblasts in order to regenerate EOMs, we hypothesized that knock-down of Twist TFs would impair EOM regeneration. Translation blocking MOs significantly decreased protein levels of all Twist TF proteins in embryos (twist1a/b and twist2) and adult EOM (twist3) (Fig 1H–1K). To test the effect of twist TF knockdown on EOM regeneration, we performed MO injections and electroporation 4 hours prior to myectomy of the right LR muscle. At 8 days post injury (dpi), control MO injected muscles were fully regrown as expected (Fig 1A–1A") [10]. The twist1a, twist1b, and twist2 MO injection groups also displayed full regeneration at 8 dpi (Fig 1B–1D"). In contrast, twist3 injected muscles at 8 dpi were significantly shorter than other groups (Fig 1E–1E" and 1F).

To determine if this effect was inhibition or just delay of regeneration, we observed the twist3 MO injected group at 21 dpi, approximately three times longer than the typical regeneration time for zebrafish LR post myectomy [10]. At 21 dpi, the length of the regenerated muscle in the twist3 knockdown group remained significantly shorter than the control group (Fig 1G), suggesting true inhibition of regeneration. Co-injection of twist1a/b, twist2, and twist3 MOs did not enhance this phenotype, suggesting that only twist3 is required for complete LR regeneration.

### Electroporation of EOMs alone does not stimulate proliferation

**Fig 2:**
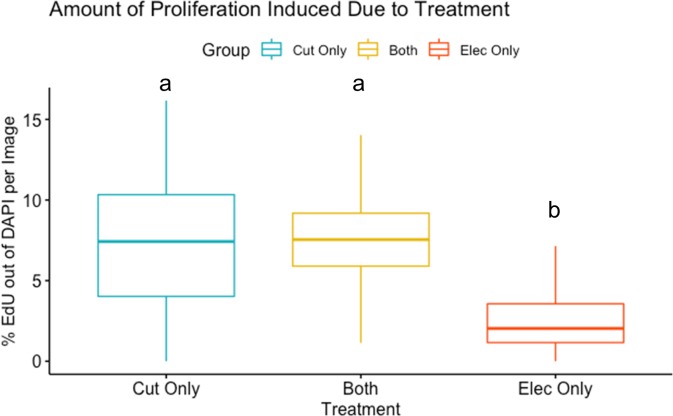
Electroporation does not cause significant damage of EOM. Boxplot of the percentage of proliferating nuclei (EdU) out of all nuclei (DAPI), in three different treatment groups: cut only (blue), both cut and electroporation (yellow), and electroporation only (red). The boxplot displays the minimum, 25^th^ percentile, median, 75^th^ percentile, and maximum for the groups. p<0.001 between two statistic groups a and b by ANOVA.

Electroporation of MOs to modify protein expression has been widely utilized in both *in vitro* and *in vivo* studies in multiple tissues [31–35]. Skeletal muscle is a favored target tissue for this technique and electroporation significantly improves the transgene efficiency [35]. However, there remains concern about muscle damage and subsequent repair associated with electroporation process [35, 36]. In order to exclude electroporation-induced damage and cellular reprogramming as a confounding variable, we assessed levels of proliferation between either electroporation or myectomy alone or in combination. We found that electroporation alone did not significantly induce cell proliferation; only ~2.5% of total myocytes were proliferating cells (EdU-positive vs DAPI-positive; Fig 2). In contrast, both cut muscle (*i*.*e*. injury only) combined treatment muscle (*i*.*e*. cut and electroporated) showed 3 times greater induction of cell proliferation compared to electroporation alone (~7.5%; Fig 2). We therefore concluded that, although electroporation does cause muscle damage and induce cellular proliferation, its extent is insignificant compared to our standard injury procedure (*i*.*e*. 50% myectomy of the lateral rectus) and does not confound the results of this study.

### Inhibition of twist3 reduces cell proliferation during muscle regeneration

Adult zebrafish EOM regeneration requires myocyte dedifferentiation, followed by a proliferative burst at 24–48 hpi [10]. After generating a sufficient number of myoblasts, cells then migrate, re-differentiate into myocytes and fuse into myotubes [10]. Based on the observed inhibition of regeneration in twist3 MO-injected fish, and the known roles of Twist TFs in stem cell biology, we hypothesized that twist3 would be important in early dedifferentiation steps leading to proliferation.

**Fig 3:**
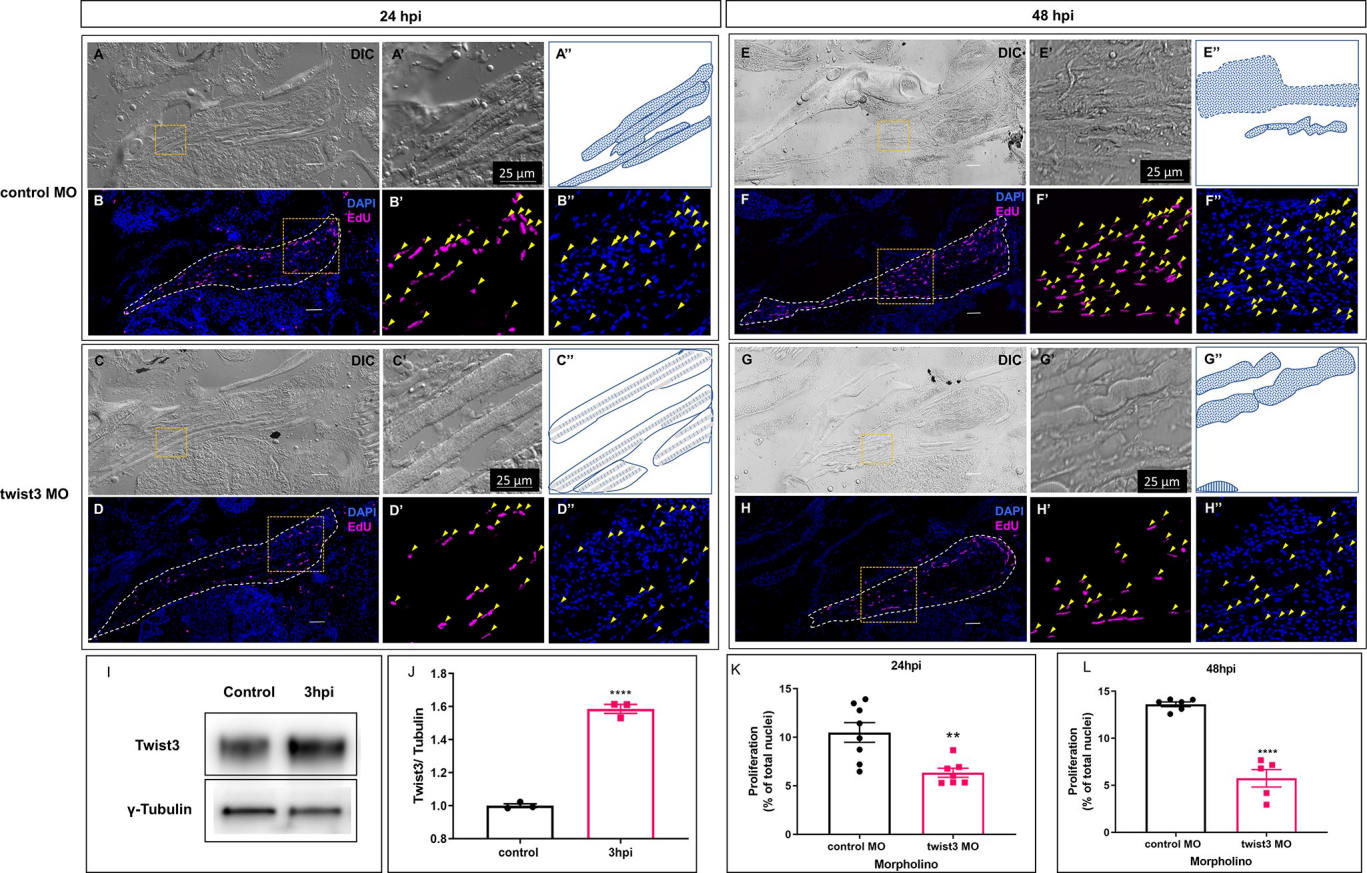
Inhibition of Twist3 impairs myocyte reprogramming and proliferation. The role of Twist3 in myocyte reprogramming and proliferation at 24 and 48 hpi was assessed by injecting Tg(*α-actin*::*EGFP*) fish with twist3 MO. Morphology of myocytes was shown using DIC images (A, C, E, G), highlighted in higher magnification (A’, C’, E’, G’) and illustrated in schematic diagram (A”, C”, E”, G”; solid line: outline of muscle; dash line” approximate outline of muscle; dotted pattern” mesenchyme-like myocytes; long light blue line: myofiber; short vertical strips: Z-band architecture). (B, D, F, H) Proliferating myoblasts were stained by EdU incorporation. EdU: magenta; DAPI: blue; Yellow arrows: positive Edu staining nuclei and corresponding DAPI channel. (I, J) Western blot analysis for Twist3 protein expression during EOM regeneration; values were averages ±SEM (t-test, p<0.0001 between control and 3hpi, n = 3). (K, L) Cell proliferation in injured muscle was significantly less in Twist3 MO injection group compared with control group at both 24 and 48 hpi. (24 hpi, control: n = 8, twist3 MO: n = 7; 48 hpi, control: n = 6, twist3 MO: n = 5) Scale bar: 50 μm, **p<0.01, ****p<0.0001.

First, we determined the timing of twist3 gene expression level by Western blot and found a 1.5-fold induction of twist3 as early as 3 hpi (Fig 3I and 3J). Next, we utilized an EdU incorporation assay to test the number of proliferating myoblasts post-injury, since proliferation of dedifferentiated myoblasts represents the final step of the reprogramming process [25, 37]. We found that the percentage of proliferating myoblasts (EdU-positive nuclei) in twist3 MO-injected fish was significantly reduced at both 24 and 48 hpi compared to control MO-injected fish (Fig 3B, 3D, 3F and 3H). In contrast, twist3 MO did not inhibit proliferation during embryonic development, since the phosphorylation of histone H3 (proliferation marker) was increased instead of decreased by twist3 MO (S1 Fig), suggesting that the role of twist3 in EOM myocyte dedifferentiation was not reflective of a general role in cell proliferation. The induction of phosphorylation of histone H3 may due to the delay of development caused by twsit3 MO injection (S2D”‘ Fig).

During regeneration of control MO-injected muscles, myofibers in the control group lost Z-band architecture and became mesenchyme-like in appearance at 24 hpi (Fig 3A’ and 3A”). It was difficult to distinguish individual myofibers and the morphology became increasingly more mesenchymal at 48 hpi (Fig 3E’ and 3E”) [10]. In contrast, in twist3 MO-injected muscles, myocytes maintained a differentiated myofiber morphology and Z-band architecture was clearly shown at 24 hpi (Fig 3C’ and 3C”). Some myofiber structure remained and Z-band could be seen in some myofibers at 48 hpi (Fig 3G’ and 3G”). Taken together, these data suggest that twist3 plays a role in the control of early myocyte reprogramming and cell cycle reentry after injury.

### Twist3 is involved in EOM regeneration via a shared pathway with Fgf

**Fig 4:**
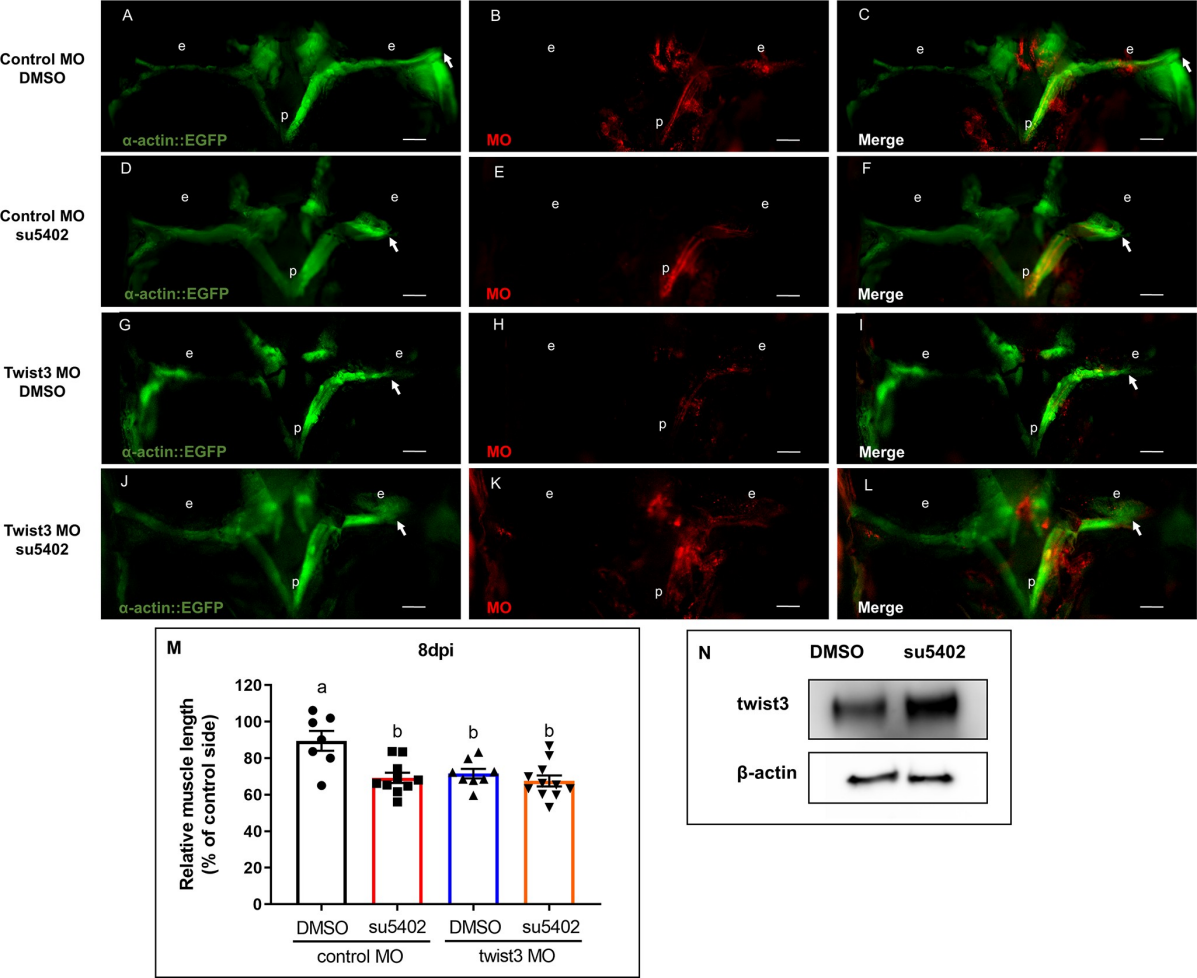
Inhibition of Fgfr and twist3 do not have an additive effect on EOM regeneration. Myectomized Tg(*α-actin*::*EGFP*) fish were treated with su5402 (D-F), or injected with twist3 MO (G-I), or treated with su5402 and injected with twist3 MO (J-L) compared with DMSO treatment and control MO injection (A-C). (M) All the experiment groups demonstrated significantly inhibited muscle regeneration, with no additive effect detected by combination of two treatments at 8 dpi. (p, pituitary; e, eye; Scale bar: 250μm, p<0.05 between two statistic groups a and b by ANOVA, control MO/DMSO: n = 7, control MO/su5402: n = 10, twist3 MO/DMSO: n = 8, twist3 MO/su5402: n = 11).

**Fig 5:**
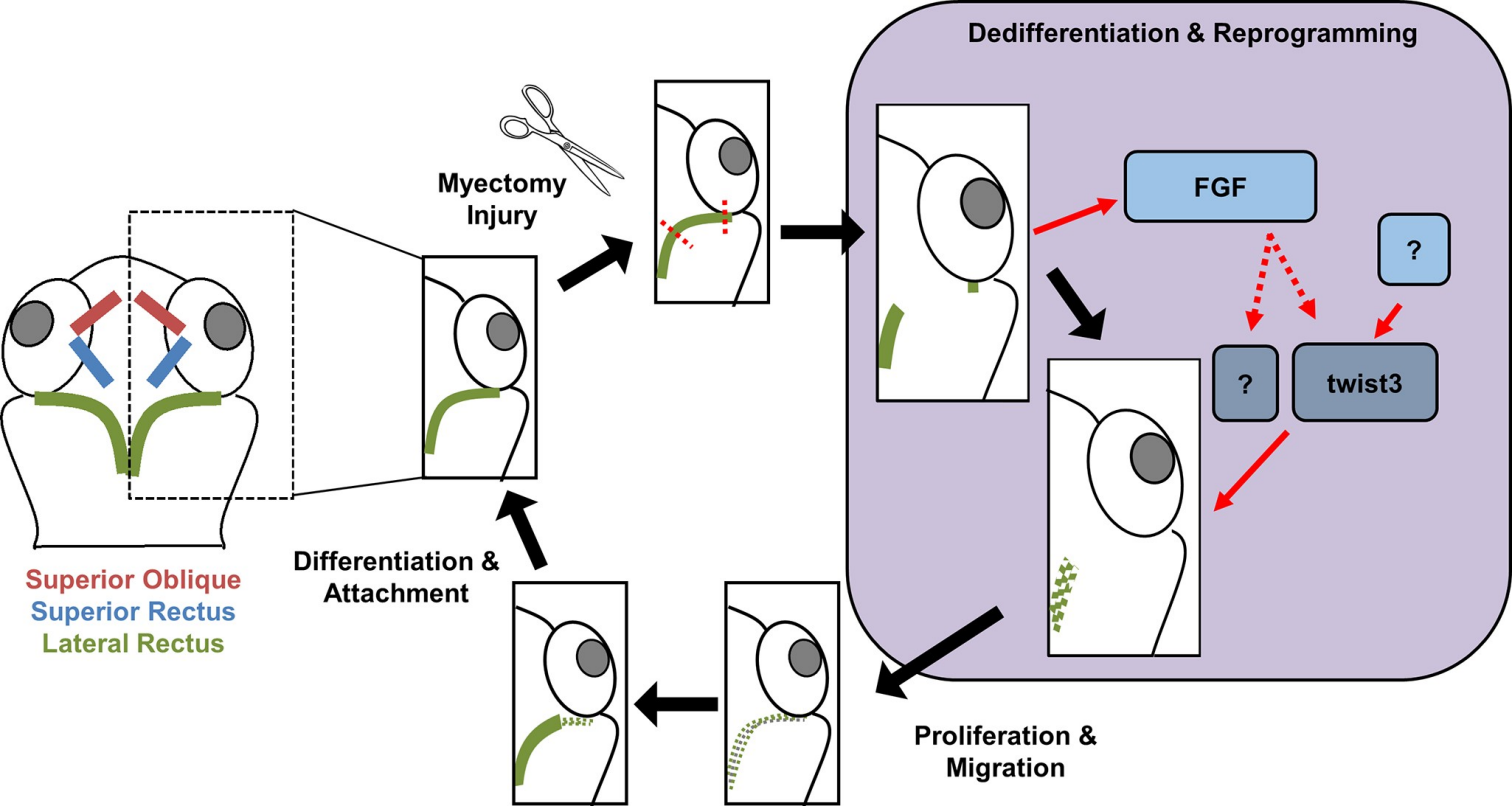
Twist3 role during EOM regeneration. Following myectomy injury, twist3 expression is elevated and promotes myocytes cell reprogramming and dedifferentiation. The known role of Fgf combined with our results (Fig 4) suggest that twist3 shares a common pathway with Fgf.

We next investigated the mechanism through which twist3 promotes EOM regeneration. Twist is required for the proper function of the Fgf-signaling pathway [38]. Our previously published study highlighted the important role of Fgf signaling in zebrafish EOM regeneration [25]. Hence, we tested the hypothesis that twist3 promotes regeneration via Fgf signaling. In order to test this hypothesis, we combined twist3 MO injection with pharmacological inhibition of Fgf using su5402, an Fgf-receptor inhibitor [25]. Both su5402 and twist3 MO injection alone significantly decreased regenerated muscle length as expected (Fig 4D–4I). Combining su5402 treatment and twist3 MO injection inhibited regeneration, but no additive/synergistic effect was observed compared to MO injection or su5402 alone (Fig 4J–4M). Twist3 protein level was higher in the su5402 group compared with the control group at 3 hpi (Fig 4N), suggesting a negative feedback loop existing between twist3 and Fgf pathways. That is, inhibition of Fgf induced the expression of twist3. In addition, the induction of twist3 could not overcome the effect of Fgf inhibition (Fig 4J–4L). Taken together, these data suggest that Fgf is an upstream—but not direct—regulator of twist3, and that Fgf has other downstream targets involved in muscle regeneration in addition of twist3 (Fig 5).

## Discussion

Cellular reprogramming from a more- to less-differentiated state requires coordinated changes in chromatin, gene expression and cellular architecture, driven by altered functionality of key transcription factors [39, 40]. Because of their role as master regulators of stem cells and in embryonic craniofacial development, we decided to use our unique zebrafish EOM regeneration model to test whether twist TFs play a role in EOM myocyte dedifferentiation.

In adult zebrafish, EOM regeneration begins with myocyte reprogramming—an EMT process—followed by cell cycle reentry, proliferation, and migration of the dedifferentiated myoblasts, and eventually re-differentiation into myocytes that fuse to form myofibers [10]. Our long-term goal is to understand regulations of cell identity and fate, by understanding the early steps of EOM myocytes reprogramming dedifferentiation.

Twist TFs belong to the basic helix-loop-helix (bHLH) family that is important for the regulation of cell fate decision and differentiation [41] and EMTs [42]. Twist TFs are also considered master regulators of stem cells in that they are important to maintaining the stem cell state [21, 29, 30]. In our regeneration model, twist TF knockdown impaired muscle regeneration by inhibiting myocyte reprogramming, revealing an early role for Twist that is consistent with the early induction of expression following injury and with Twist TF’s role in EMT. Interestingly, this effect was specific to twist3 (homolog of mammalian twist2) and none of the other paralogs, revealing evolutionary sub-functionalization in the context of adult tissue regeneration.

It has been reported that Twist is involved in adult muscle regeneration in both *Drosophila* and mice. In *Drosophila*, persistent twist expression is a marker of embryonic precursors for adult muscle [43]. Twist is also required for adult *Drosophila* myogenesis [18]. In mouse skeletal muscle, twist expression is quickly elevated after injury [20]. In addition, murine *Twist2* (an orthologue of Zebrafish *twist3*)-dependent progenitor cells contribute to muscle regeneration [21]. In adult zebrafish, twist1a and twist1b are involved in heart regeneration [44, 45]. Our study represents the first investigation of twist within adult zebrafish skeletal muscle regeneration, and our results suggest that promoting muscle regeneration may be an evolutionarily-conserved function of twist TFs.

The role of twist1in zebrafish development has been extensively studied. As EMT transcription factors, twist1 are involved in neural crest migration, which undergo an EMT to give rise to many different derivatives [46]. Regulated by thyroid hormone [47], retinoic acid (RA)[48], Wnt [49], Bmps and Id2a [28] signaling pathways, Twist 1a/b is required for proper development of craniofacial cartilage and skeleton [50], with Runx2 a known downstream target [13, 14]. Twist1 is also involved in blood vessel sprouting in zebrafish embryos [51]. Like twist1, twist2 is also involved in bone formation regulated by RA [48]. Despite their significant peptide similarity, expression locations of four twist TFs differ significantly from each other, suggesting a considerable divergence of regulatory controls [52, 53]. This is supported by our findings that different twist TFs are involved in EOM regeneration and development. Twist3 is involved in zebrafish EOM regeneration but not development. In embryos with twist3 knockdown, EOM development appeared normal, although the muscle appeared longer and thinner, possibly due to a severe bulging eye phenotype (S2D–S2D‴ Fig). EOMs also developed normally after twist1a/b knock-down (S2B–S2B" Fig). In contrast, while muscle fibers could be identified following twist2 knockdown (highlighted by actin-GFP), they failed to form a normal EOM pattern. It was difficult to differentiate the 6 pairs of EOMs based on insertion position (S2C–S2C’ Fig) compared with control fish (S2A–S2A’ Fig). Instead of normal insertion patterns, muscles seemed to “wrap around” the globe (S2C" Fig). In embryos, twist2 knockdown impaired EOM formation as early as 48 hpf (S3B–S3B" Fig). This finding reveals a key differences between zebrafish embryonic development and regeneration, suggesting that regeneration is not a simple recapitulation of developmental programs but rather a distinct program, albeit one that utilizes many of the same building blocks.

An important limitation of this study is the use of MOs to knockdown gene expression. MOs have been used widely in a variety of experimental models, such as Xenopus, zebrafish and other organisms [54]. However, in embryo research, their use has been largely supplanted by CRISPR/Cas9 genetic engineering because of concerns about MO knockdown efficiency and off-target effects [55]. It should be noted that the phenotypic differences between mutants (CRISPR/Cas9) and morphants (MO knockdown) may due to the natural activation of genetic compensation induced in mutants [56]. Nevertheless, for knocking down gene expression in select adult tissue, direct electroporation of MOs has no proper experimental substitute, and this technique has been used extensively in the adult zebrafish regeneration model [57, 58]. In this study, we followed the guidelines for use of MOs in zebrafish [55], most importantly validating multiple MOs and assessing reduction in protein level using Twist antibodies (Fig 1). Furthermore, since knockdown of different Twist family members resulted in specific phenotypic differences, our results are most consistent with a specific phenotypic effect rather than off-target effects. Ultimately, there are no alternative techniques for knocking down gene expression of a specific gene in a specific extraocular muscle, and hence electroporation of MOs represents the state of the art for these experiments.

Twist TFs are critically important to both embryonic development and cancer. The involvement of Twist in cancer includes EMT during metastasis [42, 59–61], as well as maintenance of cancer stem cells [42, 62–65]. In our study, injured EOMs in the twist3 knock-down group do not de-differentiate properly, consistent with an early role for Twist in reprogramming and EMT (i.e. muscle-to-mesenchymal transition). The similarities between cellular dedifferentiation and cancer have been previously noted [66–69], and our data provide additional supportive evidence.

## Supporting information

S1 Fig Twist3 MO does not inhibit cell proliferation during embryo development. Western blot of phospho-histone H3 shows twist3 MO injection induced p-histone H3 at 24 and 48 hpf.
(TIF)

S2 Fig Twist2 regulates EOM development in zebrafish. Tg(*α-actin*::*EGFP*) embryos that were injected with twist 1a/b, 2, or 3 MO at the one- to four-cell state demonstrated EOM formation at 5 dpf from dorsal (B-D), lateral (B'-D'), Ventral (B''-D''), and phenotype (B‴-D‴) compared with control embryos (A-A‴). SO: Superior Oblique, SR: Superior Rectus, LR: Lateral Rectus, IO: Inferior Oblique, IR: Inferior Rectus. Asterisk: undeveloped jaw muscle, red arrow: incorrectly inserted EOM, scale bar: 100μm.
(TIF)

S3 Fig Twist2 delays EOM development in zebrafish. Tg(*α-actin*::*EGFP*) embryos that were injected with twist2 MO at the one- to four-cell state demonstrated EOM formation at 48 or 72 hpf from dorsal (B, D), lateral (B'-D'), and ventral (B''-D'') compared with control embryos (A-A'', C-C''). SO: Superior Oblique, SR: Superior Rectus, LR: Lateral Rectus, IO: Inferior Oblique, IR: Inferior Rectus. Red arrow: incorrectly inserted EOM, scale bar: 100μm.
(TIF)

S1 Raw images (PDF)
