# Supplementary figures and images for: Twist3 is required for dedifferentiation during extraocular muscle regeneration in adult zebrafish
Source: PLoS One. 2020 Apr 22;15(4):e0231963. doi: 10.1371/journal.pone.0231963 (PMC7176127; doi:10.1371/journal.pone.0231963)

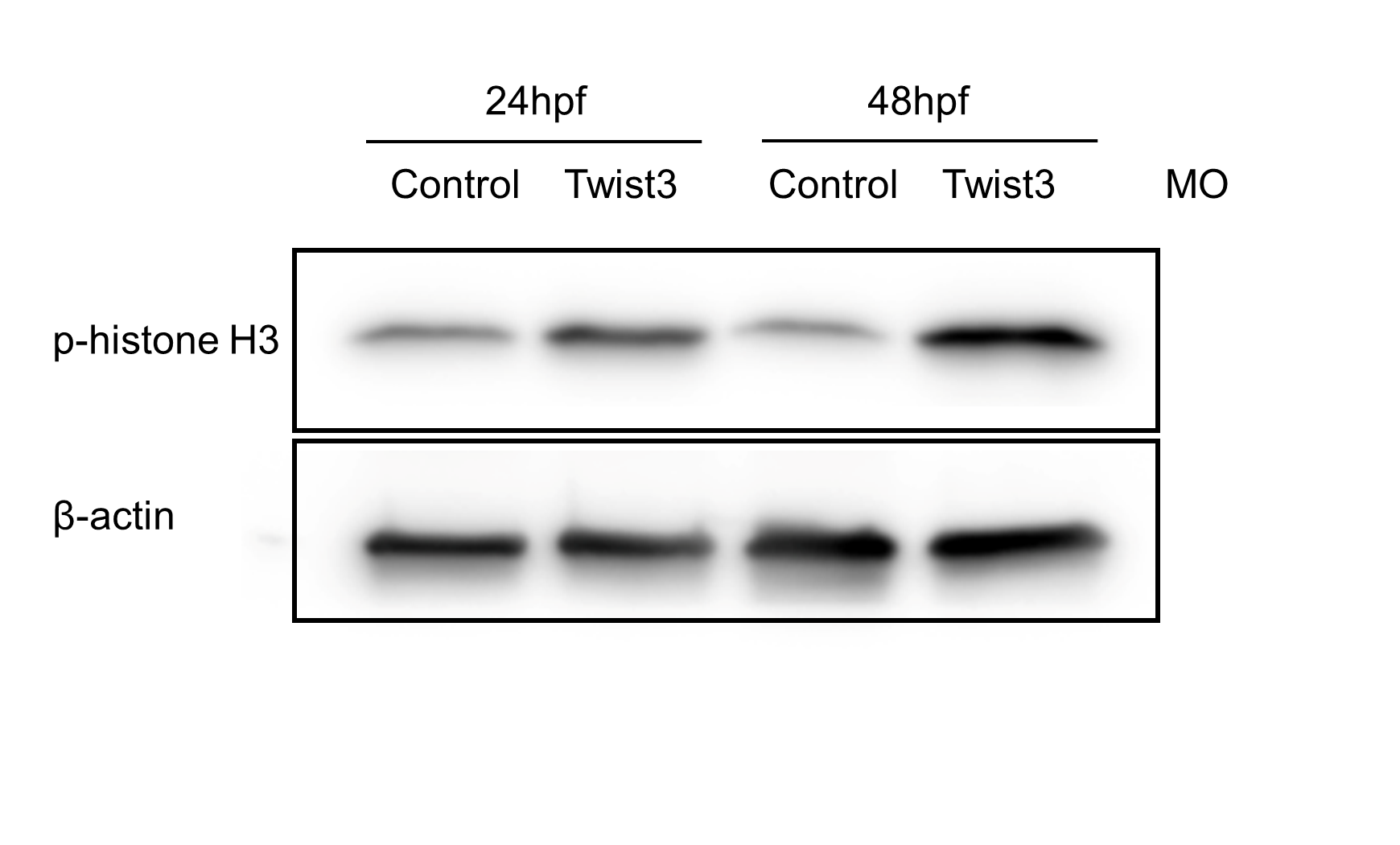

Supplement: S1 Fig — Western blot of phospho-histone H3 shows twist3 MO injection induced p-histone H3 at 24 and 48 hpf. (TIF) [file pone.0231963.s001.tif]

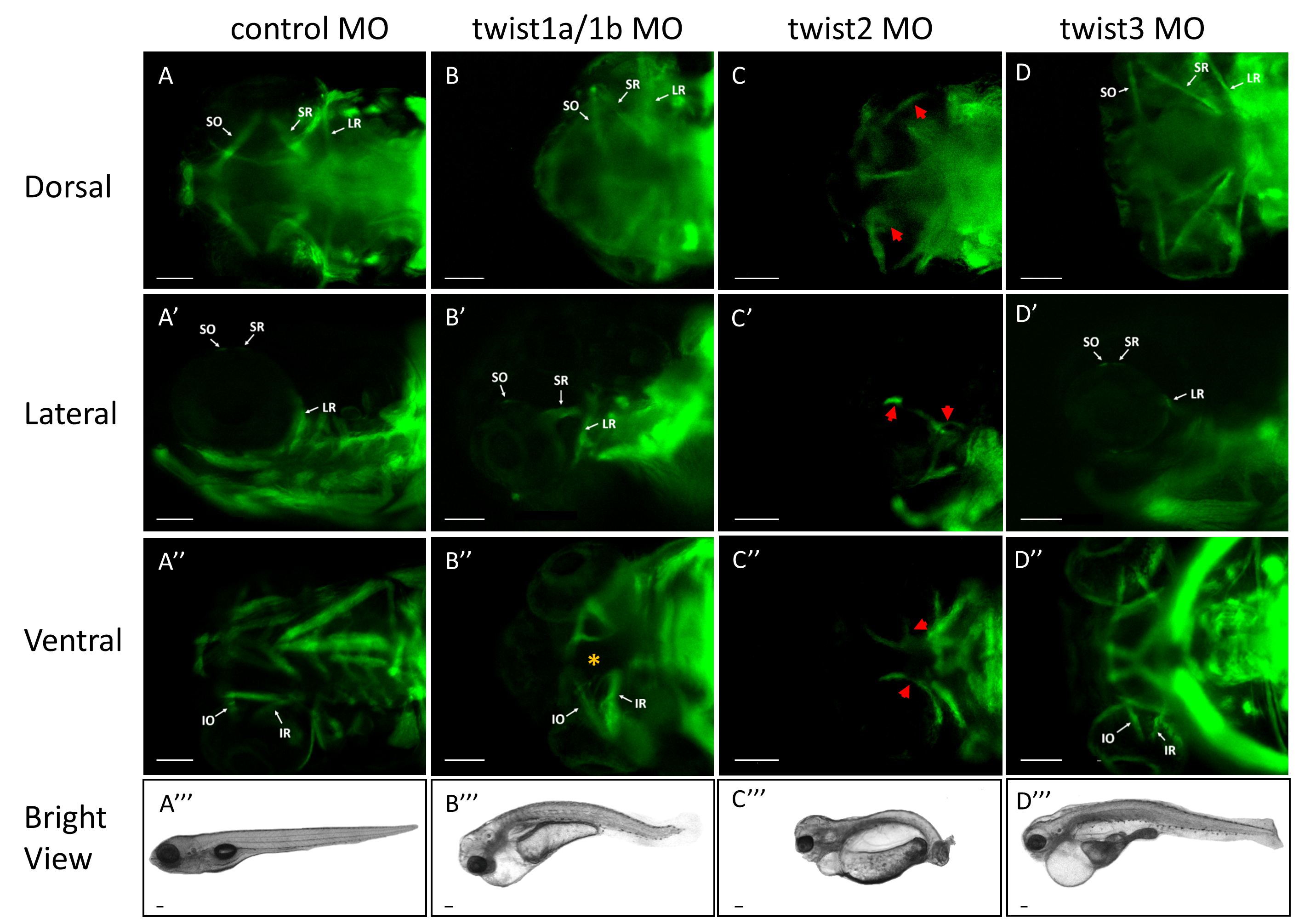

Supplement: S2 Fig — Tg(α-actin::EGFP) embryos that were injected with twist 1a/b, 2, or 3 MO at the one- to four-cell state demonstrated EOM formation at 5 dpf from dorsal (B-D), lateral (B'-D'), Ventral (B''-D''), and phenotype (B‴-D‴) compared with control embryos (A-A‴). SO: Superior Oblique, SR: Superior Rectus, LR: Lateral Rectus, IO: Inferior Oblique, IR: Inferior Rectus. Asterisk: undeveloped jaw muscle, red arrow: incorrectly inserted EOM, scale bar: 100μm. (TIF) [file pone.0231963.s002.tif]

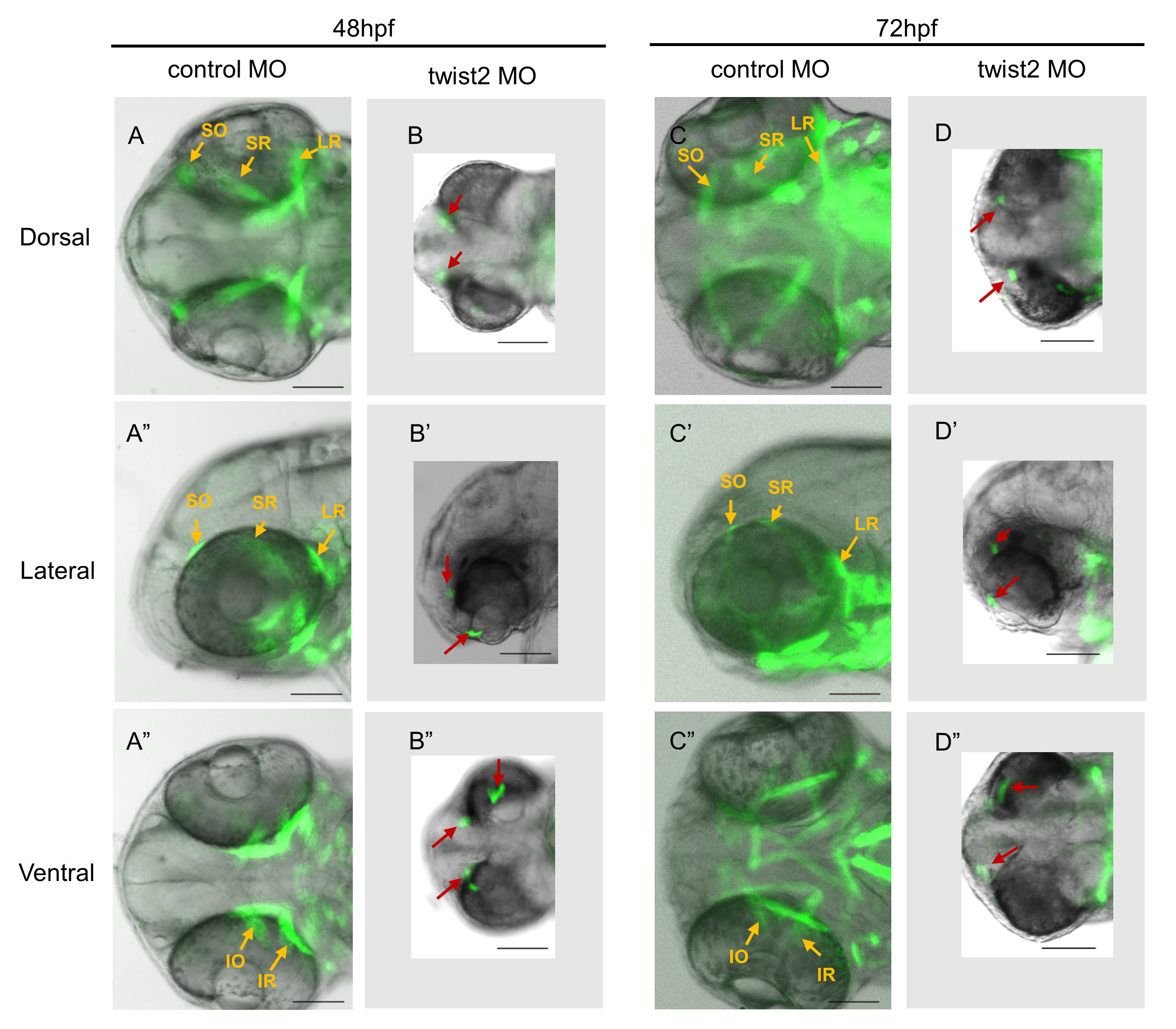

Supplement: S3 Fig — Tg(α-actin::EGFP) embryos that were injected with twist2 MO at the one- to four-cell state demonstrated EOM formation at 48 or 72 hpf from dorsal (B, D), lateral (B'-D'), and ventral (B''-D'') compared with control embryos (A-A'', C-C''). SO: Superior Oblique, SR: Superior Rectus, LR: Lateral Rectus, IO: Inferior Oblique, IR: Inferior Rectus. Red arrow: incorrectly inserted EOM, scale bar: 100μm. (TIF) [file pone.0231963.s003.tif]
