# Supplementary material for: Twist3 is required for dedifferentiation during extraocular muscle regeneration in adult zebrafish
Source: PLoS One. 2020 Apr 22;15(4):e0231963. doi: 10.1371/journal.pone.0231963 (PMC7176127; doi:10.1371/journal.pone.0231963)
Supplement: S1 Raw images — (PDF) [file pone.0231963.s004.pdf]

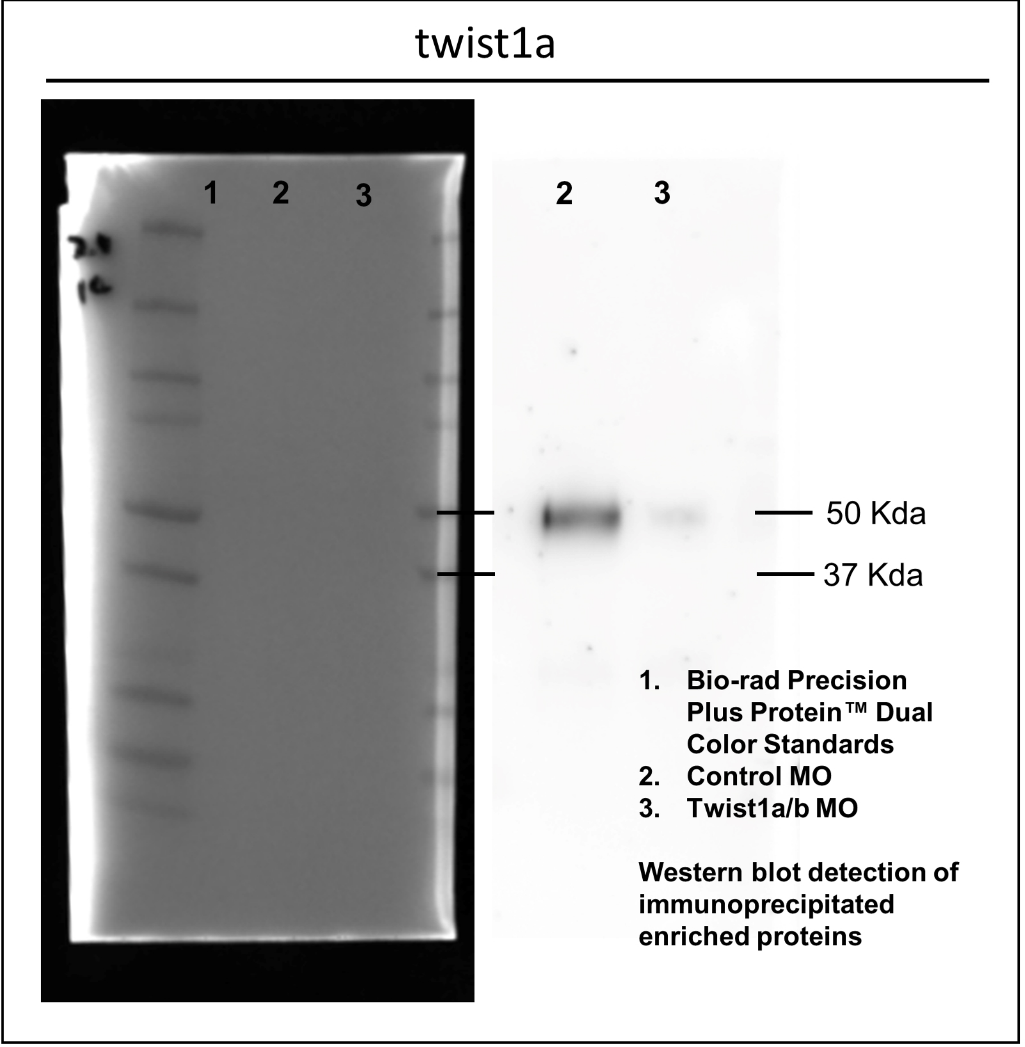
twist1a
1
2
3
2
3
50 Kda
37 Kda
1. Bio-rad Precision Plus Protein™ Dual Color Standards
2. Control MO
3. Twist1a/b MO
Western blot detection of immunoprecipitated enriched proteins

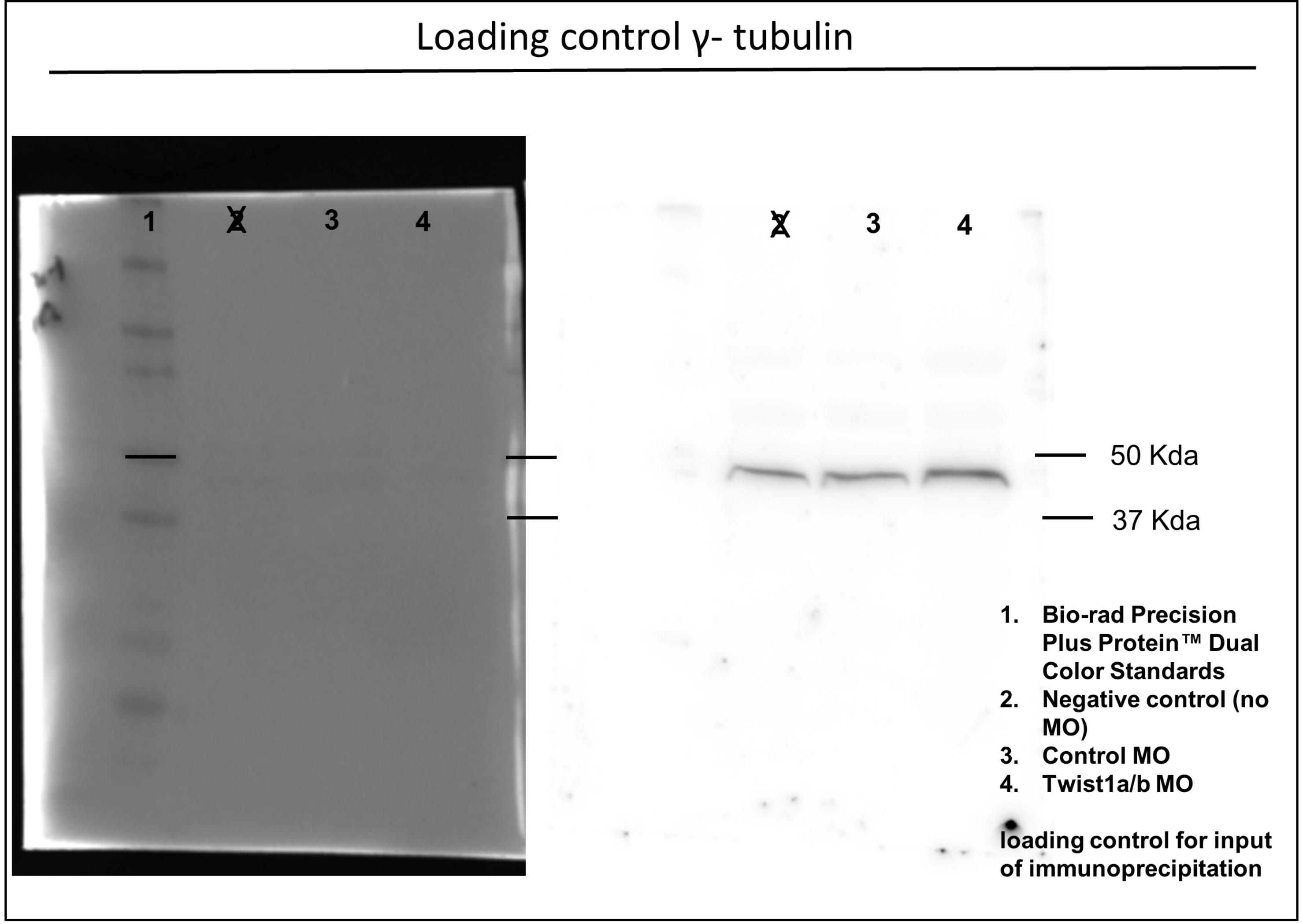
Loading control γ- tubulin
1
2
3
4
2
3
4
50 Kda
37 Kda
1. Bio-rad Precision Plus Protein™ Dual Color Standards
2. Negative control (no MO)
3. Control MO
4. Twist1a/b MO
loading control for input of immunoprecipitation

Figure 1H

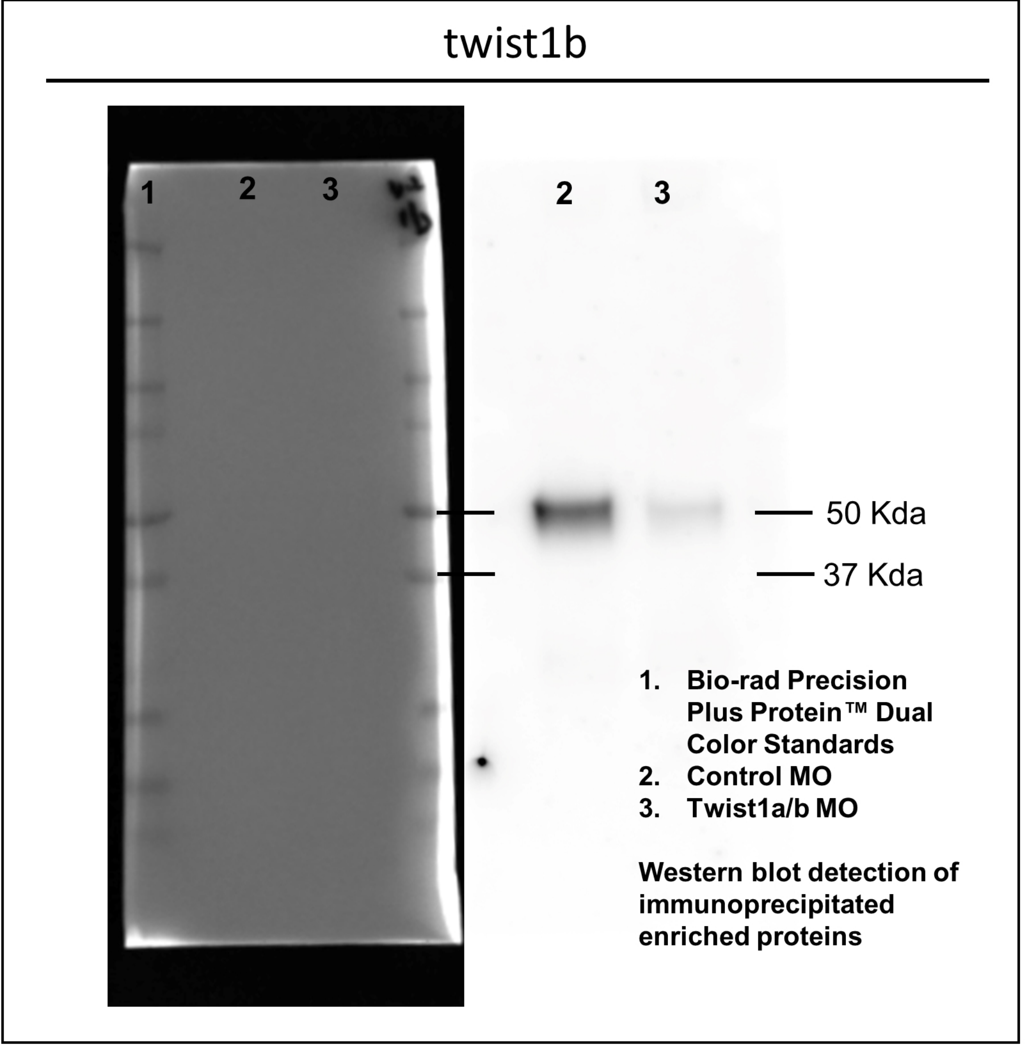
twist1b
1
2
3
2
3
50 Kda
37 Kda
1. Bio-rad Precision Plus Protein™ Dual Color Standards
2. Control MO
3. Twist1a/b MO
Western blot detection of immunoprecipitated enriched proteins

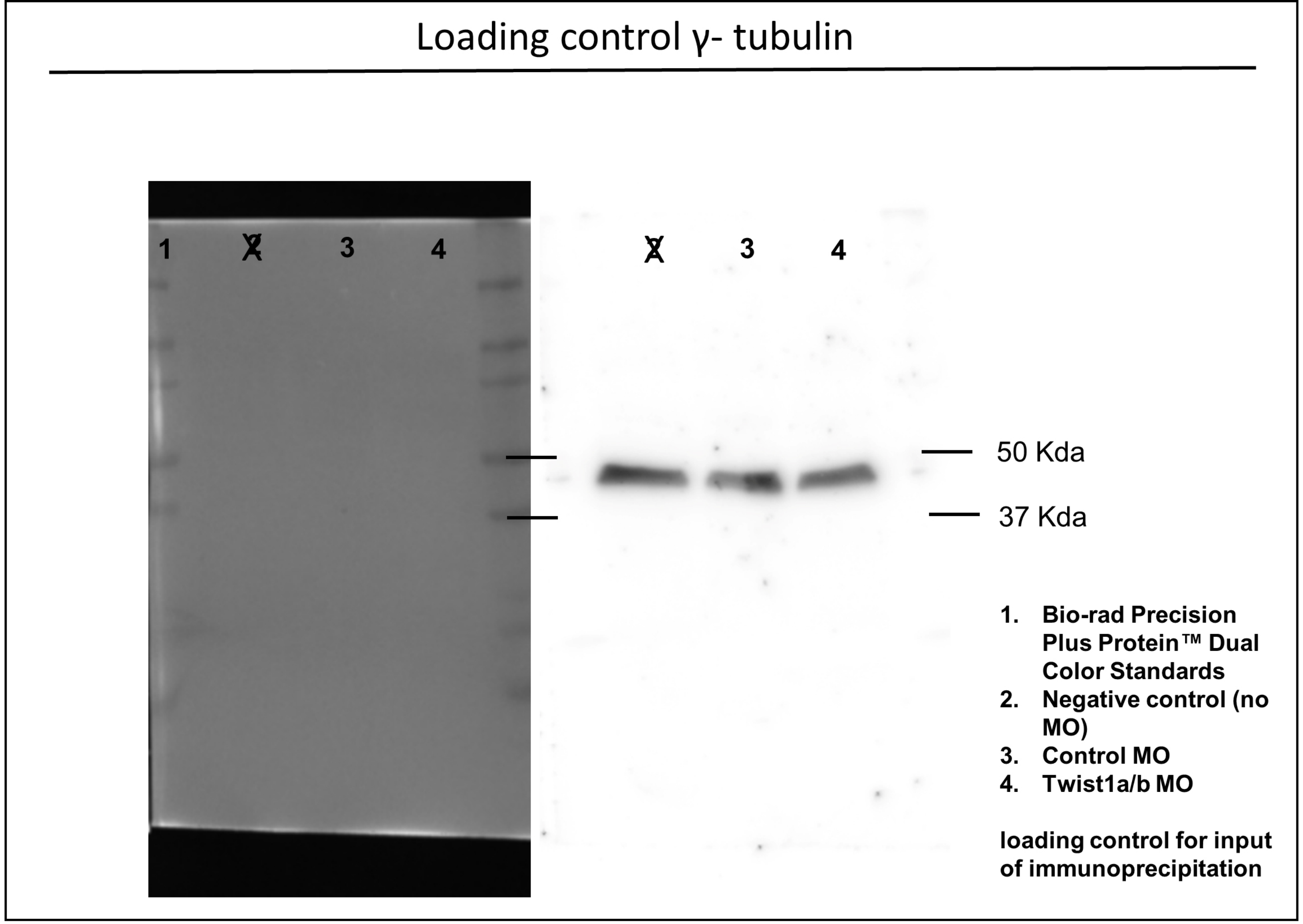
Loading control γ- tubulin
1
2
3
4
2
3
4
50 Kda
37 Kda
1. Bio-rad Precision Plus Protein™ Dual Color Standards
2. Negative control (no MO)
3. Control MO
4. Twist1a/b MO
loading control for input of immunoprecipitation

Figure 1l

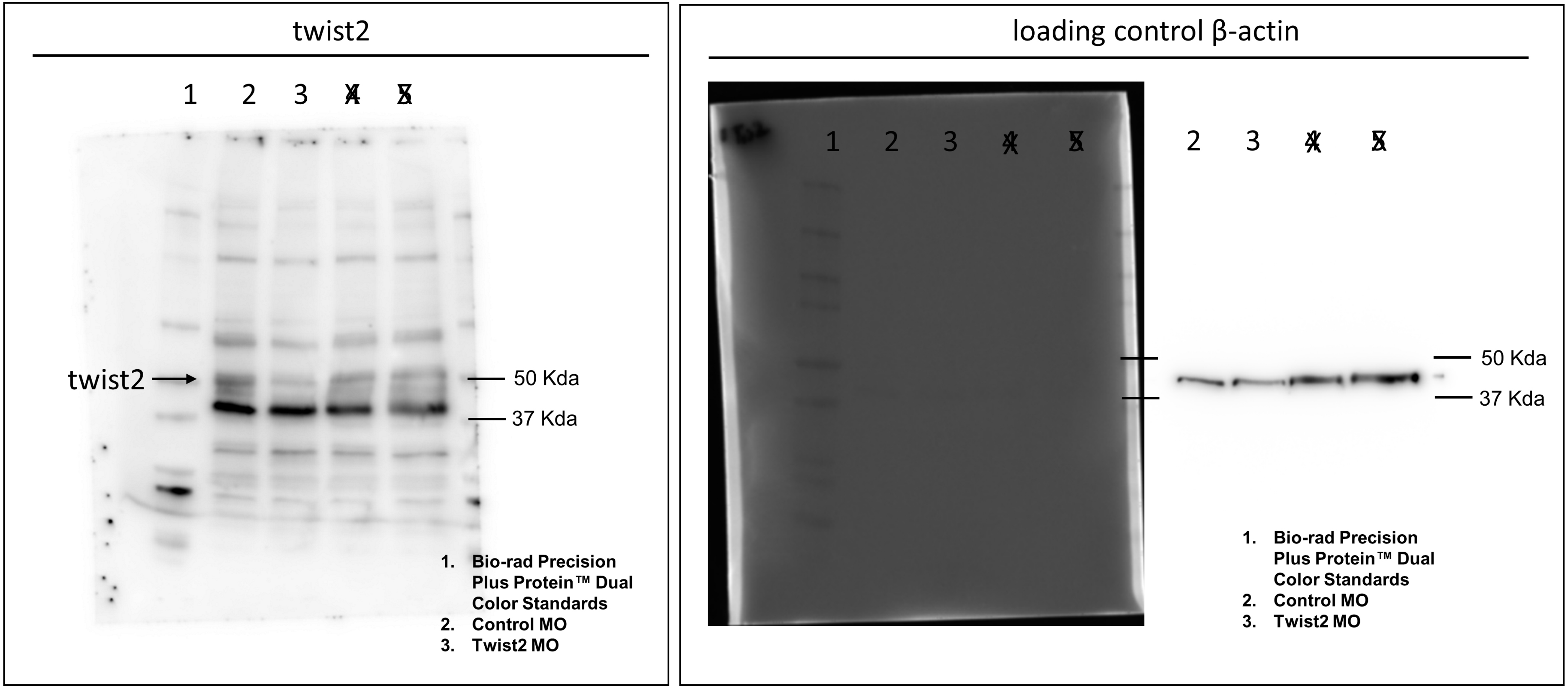
twist2
1 2 3 4 5
twist2
50 Kda
37 Kda
1. Bio-rad Precision Plus Protein™ Dual Color Standards
2. Control MO
3. Twist2 MO
loading control β-actin
1 2 3 4 5
2 3 4 5
50 Kda
37 Kda
1. Bio-rad Precision Plus Protein™ Dual Color Standards
2. Control MO
3. Twist2 MO

Figure 1J

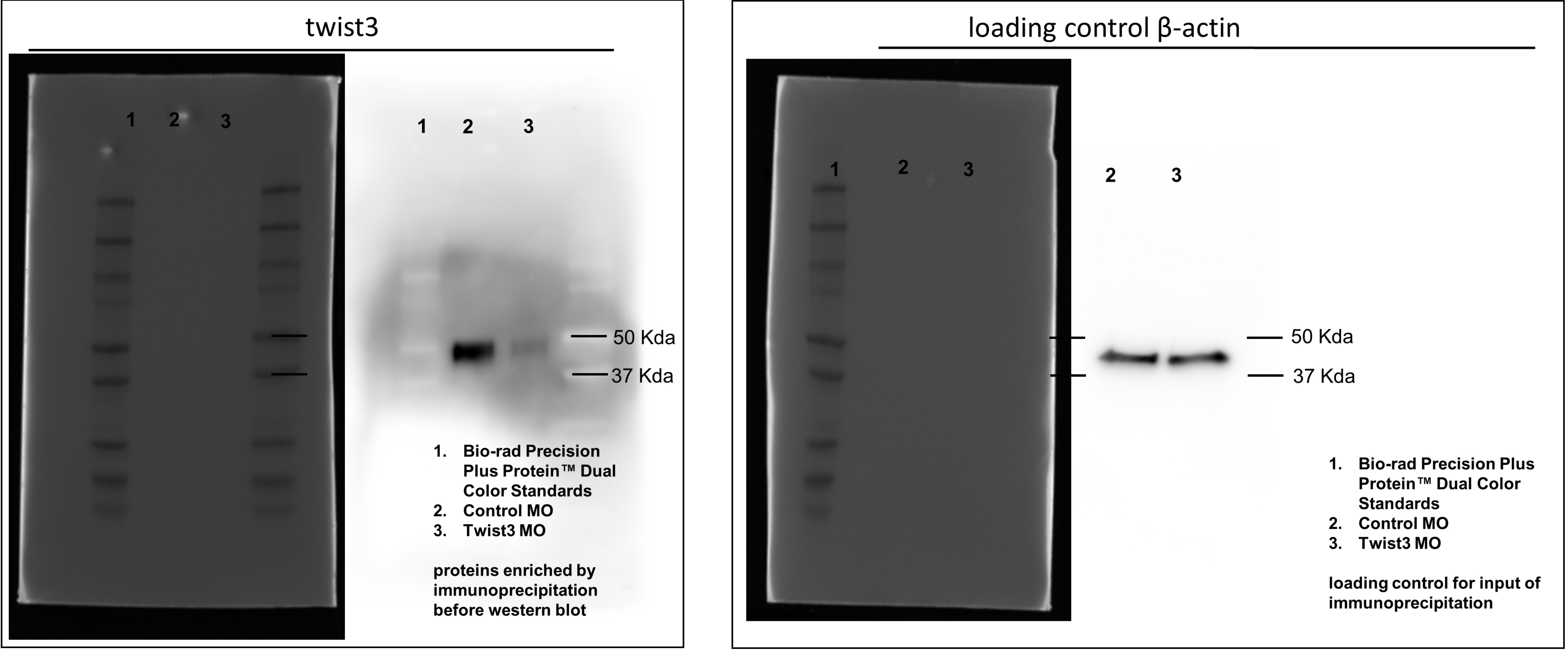
twist3
1 2 3
1 2 3
50 Kda
37 Kda
1. Bio-rad Precision Plus Protein™ Dual Color Standards
2. Control MO
3. Twist3 MO
proteins enriched by immunoprecipitation before western blot
loading control β-actin
1 2 3
2 3
50 Kda
37 Kda
1. Bio-rad Precision Plus Protein™ Dual Color Standards
2. Control MO
3. Twist3 MO
loading control for input of immunoprecipitation

Figure 1K

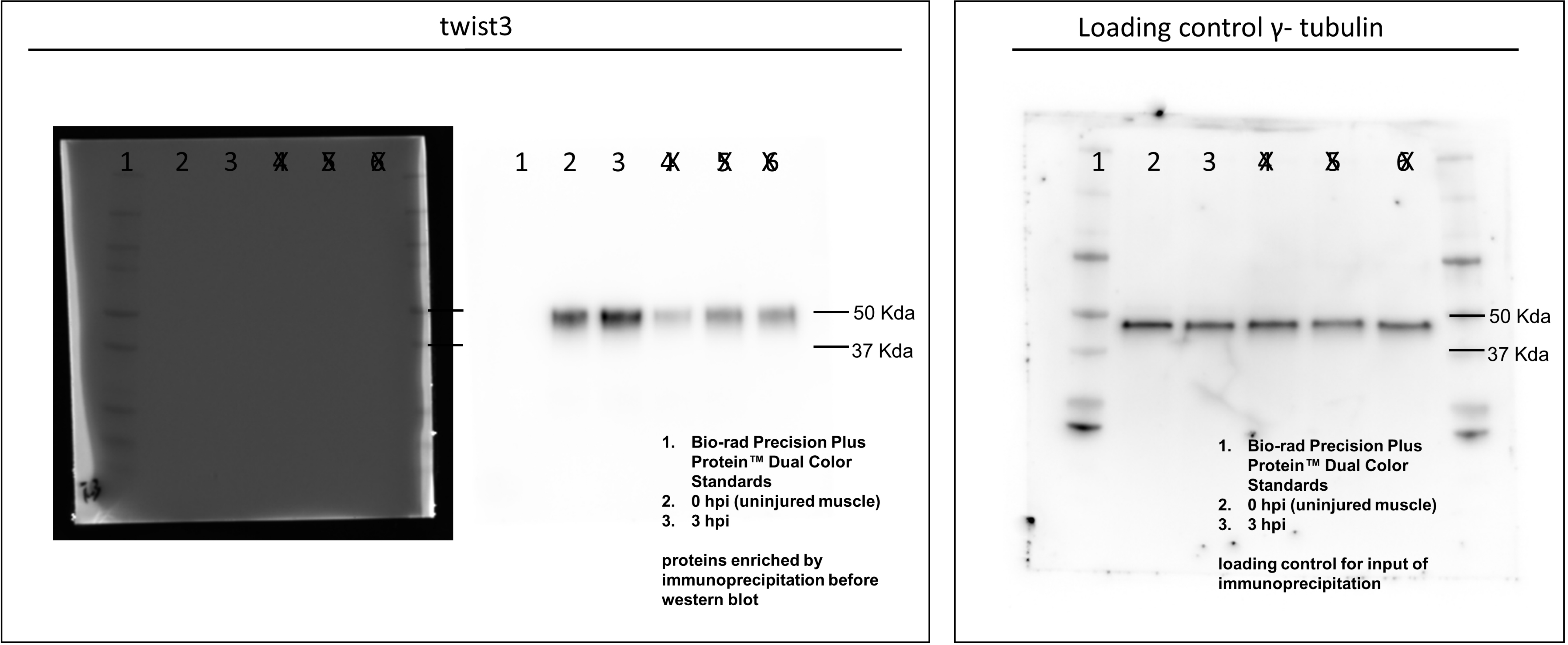
twist3
1 2 3
50 Kda
37 Kda
1. Bio-rad Precision Plus Protein™ Dual Color Standards
2. 0 hpi (uninjured muscle)
3. 3 hpi
proteins enriched by immunoprecipitation before western blot
Loading control γ- tubulin
1 2 3
50 Kda
37 Kda
1. Bio-rad Precision Plus Protein™ Dual Color Standards
2. 0 hpi (uninjured muscle)
3. 3 hpi
loading control for input of immunoprecipitation

Figure 3I

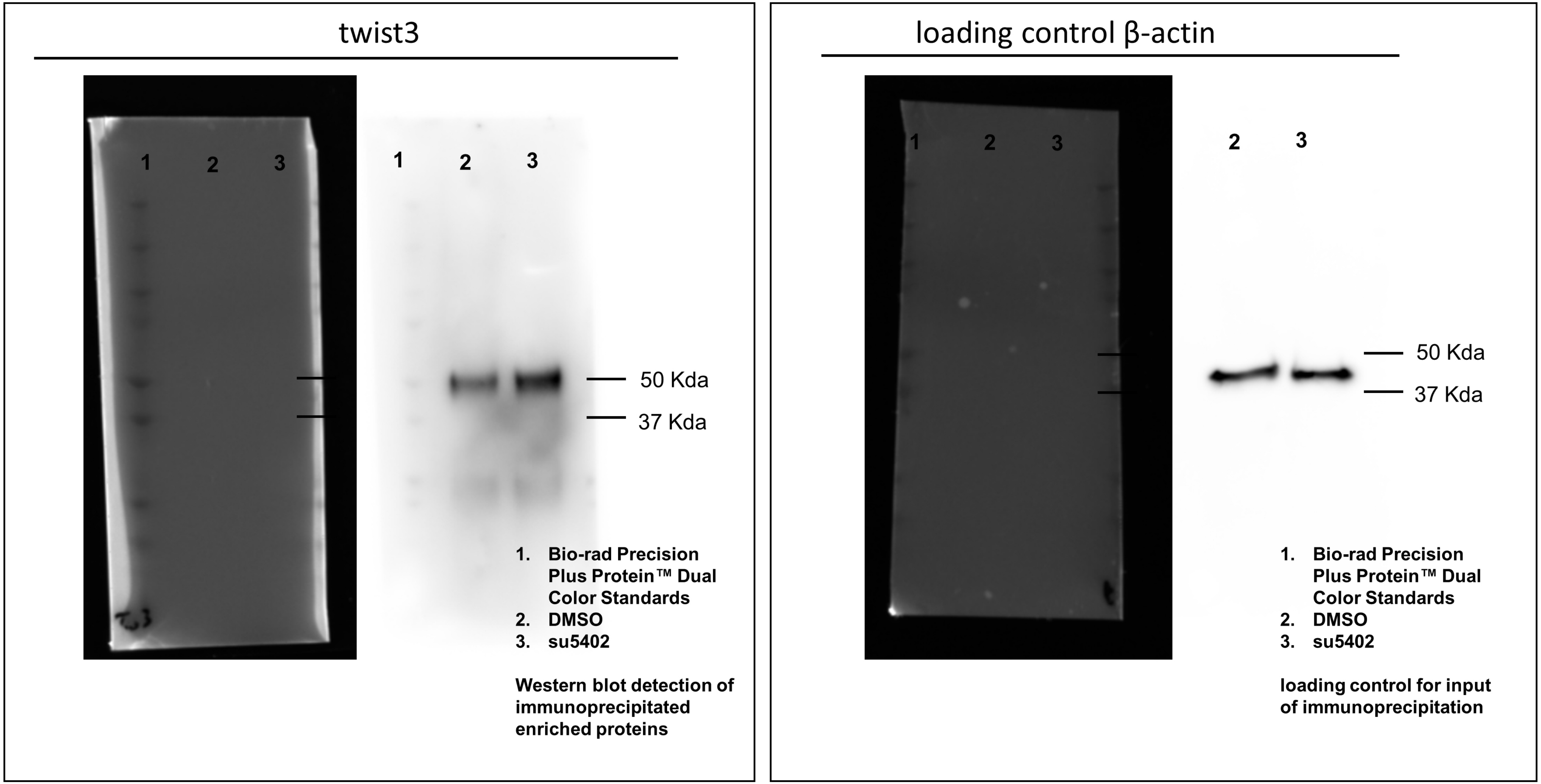
twist3
1
2
3
50 Kda
37 Kda
1. Bio-rad Precision Plus Protein™ Dual Color Standards
2. DMSO
3. su5402
Western blot detection of immunoprecipitated enriched proteins
loading control β-actin
1
2
3
50 Kda
37 Kda
1. Bio-rad Precision Plus Protein™ Dual Color Standards
2. DMSO
3. su5402
loading control for input of immunoprecipitation

Figure 4N

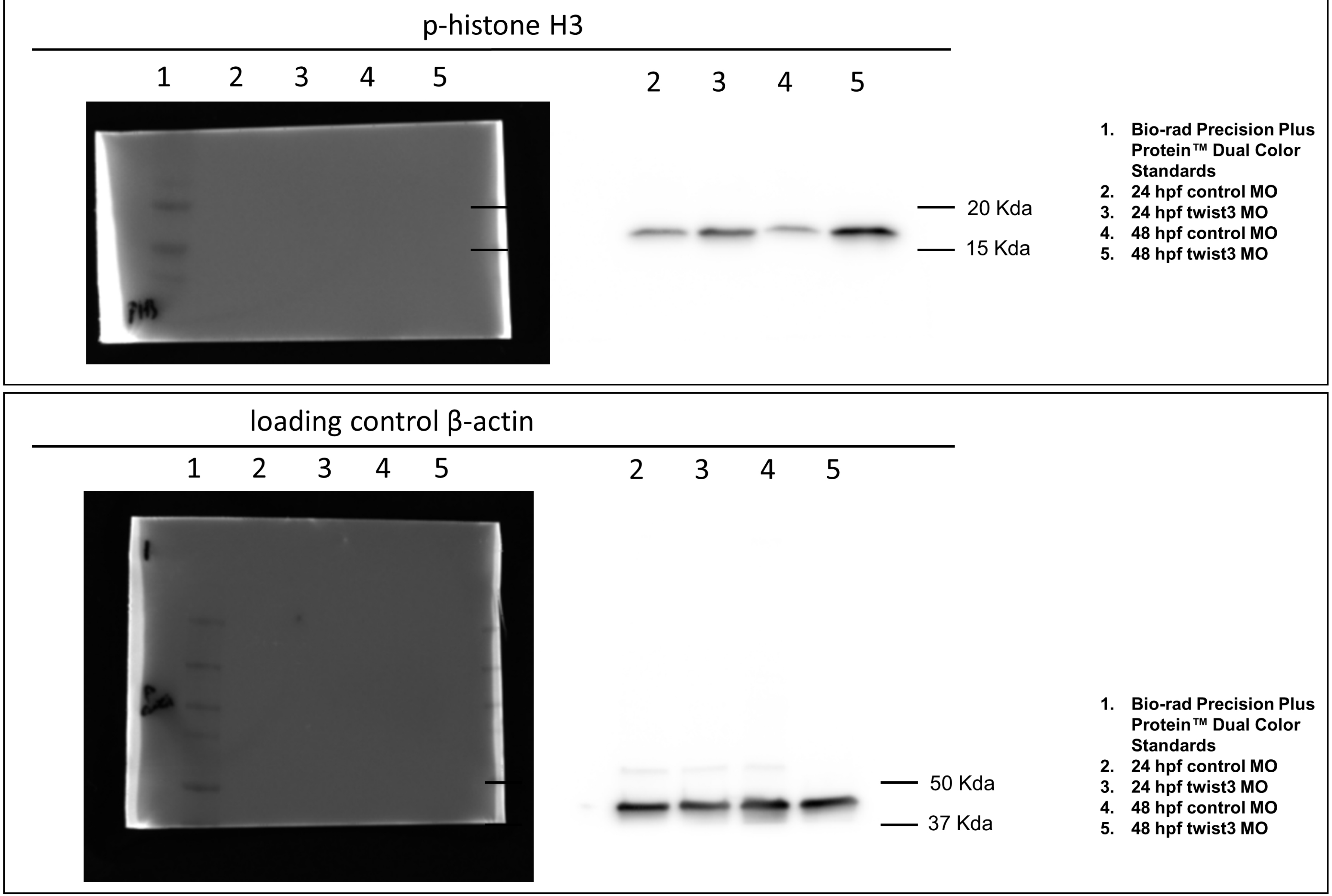
p-histone H3
1 2 3 4 5
2 3 4 5
20 Kda
15 Kda
1. Bio-rad Precision Plus Protein™ Dual Color Standards
2. 24 hpf control MO
3. 24 hpf twist3 MO
4. 48 hpf control MO
5. 48 hpf twist3 MO
loading control β-actin
1 2 3 4 5
2 3 4 5
50 Kda
37 Kda
1. Bio-rad Precision Plus Protein™ Dual Color Standards
2. 24 hpf control MO
3. 24 hpf twist3 MO
4. 48 hpf control MO
5. 48 hpf twist3 MO

Sup Figure 1
